# Supplementary material for: Developmental assembly of multi-component polymer systems through interconnected synthetic gene networks in vitro
Source: Nat Commun. 2024 Oct 3;15:8561. doi: 10.1038/s41467-024-52986-z (PMC11452209; doi:10.1038/s41467-024-52986-z)
Supplement: Supplementary file 1 — Supplementary Information [file 41467_2024_52986_MOESM1_ESM.pdf]

Supplementary Information

Developmental assembly of multi-component polymer systems through interconnected gene networks *in vitro*

Daniela Sorrentino,[1,2] Simona Ranallo,[2] Francesco Ricci,[2,*] and Elisa Franco,[1,*]

[1] Department of Mechanical and Aerospace Engineering and of Bioengineering, University of California at Los Angeles, 420 Westwood Plaza, Los Angeles, California 90095, United States.

[2] Department of Chemical Sciences and Technologies, University of Rome, Tor Vergata, Via della Ricerca Scientifica, 00133 Rome, Italy.

Supplementary Tables: oligonucleotide sequences **2**
Table 1: Green system, Tile 1 2
Table 2: Red system, Tile 2 3
Table 3: Blue system, Tile 3 3
Table 4: High yield design 4
Table 5. Connector system for high yield design 4
Table 6. Modified sequences for high yield design 5
Table 7: Low yield design 6
Table 8. Connector system for low yield design 6
Table 9. Modified sequences for low yield design 6
Supplementary Figures **7**
Green system: Tile 1 7
Red system: Tile 2 7
Blue system: Tile 3 8
High yield genes for DNA tile activation 9
Low yield genes for DNA tile activation 10
High yield genes for DNA tile inhibition 11
Supplementary Note 1: Data fitting **27**
Supplementary Note 2: Modeling **27**
References **30**

Supplementary Tables: oligonucleotide sequences

Building on previous work, we have re-engineered three different DNA tiles (green, red, and blue) that contain the same 5-nt sticky ends portion responsible for self-assembly but differ in the toehold sequence that can be orthogonally targeted by different regulatory strands.[3,4] A stock solution of DNA tiles was first formed from five strands (referred to as S1-S5) at a concentration of 5 µM and annealed with a Bio-Rad Mastercycler Gradient Thermal Cycler by heating to 90 °C and cooling to 25 °C at a constant rate over a period of 6 hours.

In addition to the DNA sequences, this section also includes schematic representations of each tile type. Nucleotides in *italics* for strands S2 and S4 denote the sticky end portions. Strand S3 was conjugated to a fluorophore at the 5' end. Green, red, or blue circles on the blue strand (S3) represent the position of the Cy3, Cy5, or 6- FAM fluorophores, as indicated in the strand sequence. Strand S2 also contains the 7-nt inhibitor binding domain (**bold**). The inhibitor strand binds to S2 via a 14-nt portion that first binds to the 7-nt inhibitor-binding domain of S2 and then invades the 5-nt sticky end and 2 additional nucleotides. The inhibitor strand contains an additional 6-nt portion (<u>underlined</u>) that remains available for binding of the activator strand. The activator first binds to this portion and then invades the duplex formed from the inhibitor and S2 strand, displacing the inhibitor from the tile and restoring the tile's ability to self-assemble.

<u>Supplementary Table 1: Green system, Tile 1</u>

**Name**	**Sequence**
S1	5'- CTC AGT GGA CAG CCG TTC TGG AGC GTT GGA CGA AAC T
S2	5'- **TGG TAT T** *GTC TG* GTA GAG CAC CAC TGA G *AGG TA*
S3 (G)	5' - (Cy3) -TCC AGA ACG GCT GTG GCT AAA CAG TAA CCG AAG CAC CAA CGC T
S4	5' - *CAGAC* AG TTT CGT GGT CAT CGT ACC T
S5	5' - CGA TGA CCT GCT TCG GTT ACT GTT TAG CCT GCT CTA C
DNA Inhibitor (G)	5' - ACC AGA CAA TAC CA <u>ATC CGC</u>
RNA Inhibitor (G)	5' - ACC AGA CAA UAC CA <u>AUC CGC</u>
DNA Activator (G)	5'- <u>GCG GAT</u> TGG TAT TGT CTG GT
RNA Activator (G)	5'- <u>GCG GAU</u> UGG UAU UGU CUG GU

Supplementary Table 2: Red system, Tile 2

Name	Sequence
S1	5'- CTC AGT GGA CAG CCG TTC TGG AGC GTT GGA CGA AAC T
S2	5'- **CTT ACG T** *GTC TG* GTA GAG CAC CAC TGA G *AGG TA*
S3 (R)	5' - (Cy5) -TCC AGA ACG GCT GTG GCT AAA CAG TAA CCG AAG CAC CAA CGC T
S4	5' - *CAGAC* AG TTT CGT GGT CAT CGT ACC T
S5	5' - CGA TGA CCT GCT TCG GTT ACT GTT TAG CCT GCT CTA C
DNA Inhibitor (R)	5' - ACC AGA CAC GTA AGG ATG GC
RNA Inhibitor (R)	5' - ACC AGA CAC GUA AGG AUG GC
DNA Activator (R)	5'- GCC ATC CTT ACG TGT CTG GT
RNA Activator (R)	5'- GCC AUC CUU ACG UGU CUG GU

Supplementary Table 3: Blue system, Tile 3

Name	Sequence
S1	5'- CTC AGT GGA CAG CCG TTC TGG AGC GTT GGA CGA AAC T
S2	5'- **AGT TCA A** *GTC TG* GTA GAG CAC CAC TGA G *AGG TA*
S3 (B)	5' - (6-FAM) -TCC AGA ACG GCT GTG GCT AAA CAG TAA CCG AAG CAC CAA CGC T
S4	5' - *CAGAC* AG TTT CGT GGT CAT CGT ACC T
S5	5' - CGA TGA CCT GCT TCG GTT ACT GTT TAG CCT GCT CTA C
DNA Inhibitor (B)	5' - ACC AGA CTT GAA CTT CGA CC
RNA Inhibitor (B)	5' - ACC AGA CUU GAA CUU CGA CC
DNA Activator (B)	5'- GGT CGA AGT TCA AGT CTG GT
RNA Activator (B)	5'- GGU CGA AGU UCA AGU CUG GU

In the following sequences, the underlined portion represents the promoter site recognized by T7 RNA polymerase, while the *italics* domain represents the portion that is transcribed. The genes contain "sealing" domains in the genes at the 5' end of the non-template strand to

prevent breathing at the promoter site. To ensure good transcriptional yield, each strand was designed to begin with G. Hence one **G** (**bold**) was added after the promoter sequence.

Supplementary Table 4: High yield design

**Gene**	**Sequence**
NonTemplate Activator 1	5'- G CGC TAA TAC GAC TCA CTA TA **G** *GCG GAT TGG TAT TGT CTG GT*
Template Activator 1	5'- ACC AGA CAA TAC CAA TCC GCC TAT AGT GAG TCG TAT TAG CGC
NonTemplate Activator 2	5'- CAA TAC CAA TCC GC TAA TAC GAC TCA CTA TA **G** *GCC ATC CTT ACG TGT CTG GT*
Template Activator 2	5' - ACC AGA CAC GTA AGG ATG GCC TAT AGT GAG TCG
NonTemplate Activator 3	5' – CAC GTA AGG ATG GC TAA TAC GAC TCA CTA TA **G** *GGT CGA AGT TCA AGT CTG GT*
Template Activator 3	5' - ACC AGA CTT GAA CTT CGA CCC TAT AGT GAG TCG
NonTemplate Inhibitor 1	5'- CT TGA ACT TCG ACC TAA TAC GAC TCA CTA TA **G** *ACC AGA CAA TAC CA ATC CGC*
Template Inhibitor 1	5'- GCG GAT TGG TAT TGT CTG GTC TAT AGT GAG TCG
NonTemplate Inhibitor 2	5'- TGG TAT TGT CTG GT TAA TAC GAC TCA CTA TA **G** *ACC AGA CAC GTA AGG ATG GC*
Template Inhibitor 2	5'- GCC ATC CTT ACG TGT CTG GTC TAT AGT GAG TCG
NonTemplate Inhibitor 3	5'- CTT ACG TGT CTG GT TAA TAC GAC TCA CTA TA **G** *ACC AGA CTT CGA CC*
Template Inhibitor 3	5'- GGT CGA AGT CTG GTC TAT AGT GAG TCG

Supplementary Table 5. Connector system for high yield design

**Connector system**	**Sequence**
Released Strand 1_6nt Toehold	5'- TAA TA GCG TGG TAT TG
Non Released Strand 1	5'- ACC AGA CA ATA CCA ATC CGC
Released Strand 2_6nt Toehold	5'- TAT TA GCC ATC CTT ACG TG
Non Released Strand 2	5' – ACC AGA CA CGT AAG GAT GGC
Released Strand 3_6nt Toehold	5'- TAT TA GGT CGA AGT TCA AG

Released Strand 3_5nt Toehold	5’- TAT TA GGT CGA AGT TCA AGT
Released Strand 3_4nt Toehold	5’- TAT TA GGT CGA AGT TCA AGT C
Released Strand 3_3nt Toehold	5’- TAT TA GGT CGA AGT TCA AGT CT
Non Released Strand 3	5’ - ACC AGA CT TGA ACT TCG ACC
Released Strand 4_6nt Toehold	5’- TAT TA ACC AGA CAA TAC CA
Released Strand 4_ 5nt Toehold	5’- TAT TA ACC AGA CAA TAC CAA
Released Strand 4_4nt Toehold	5’- TAT TA ACC AGA CAA TAC CAA T
Released Strand 4_3nt Toehold	5’- TAT TA ACC AGA CAA TAC CAA TC
Non Released Strand 4	5’- GCG GAT TG GTA GTT TCT GGT
Released Strand 5_6nt Toehold	5’- TAT TA ACC AGA CAC GTA AG
Release Strand 5_5nt Toehold	5’- TAT TA ACC AGA CAC GTA AGG
Released Strand 5_4nt Toehold	5’- TAT TA ACC AGA CAC GTA AGG A
Released Strand 5_3nt Toehold	5’- TAT TA ACC AGA CAC GTA AGG AT
Non Released Strand 5	5’- GCC ATC CT TAC GTG TCT GGT

Supplementary Table 6. Modified sequences for high yield design

To study the strand displacement reaction that occurs after the RNA activator is produced during gene transcription, we used the following sequences modified with fluorophores and quenchers.

**Modified strands**	**Sequence**
Released Strand 1	5’- TAT TA GCG TGG TAT TG - (Cy3)
Non Released Strand 1	5’- (BHQ-1) - ACC AGA CA ATA CCA ATC CGC

Released Strand 2	5'- TAT TA GCC ATC CTT ACG TG - (Cy5)
Non Released Strand 2	5'- (BHQ-2)- ACC AGA CA CGT AAG GAT GGC
S2 (G)_Q	5'- TGG TAT T GTC TG GT – (BHQ-1) - A GAG CAC CAC TGA G AGG TA
S2 (R)_Q	5'- CTT ACG TGTC TG GT – (BHQ-2) – A GAG CAC CAC TGA G A GG TA
RNA Inhibitor (G)_F	5'- (Cy3) - ACC AGA CAA UAC CA AUC CGC
RNA Inhibitor (R)_F	5'- (Cy5) - ACC AGA CAC GUA AGG AUG GC

Supplementary Table 7: Low yield design

**Gene**	**Sequence**
NonTemplate Activator 1	5'- G CGC TAA TAC GAC TCA CTA TA **G** *GCG GAT TGG TAT TGT CTG GT*
Template Activator 1	5'- ACC AGA CAA TAC CAA TCC GCC TAT AGT GAG TCG TAT TAG CGC
NonTemplate Activator 2	5'- C GAC TCA CTA TA **G** *GCC ATC CTT ACG TGT CTG GT*
Template Activator 2	5'- ACC AGA CAC GTA AGG ATG GCC TAT AGT GAG TCG GTA TTA CCA GAC AAT ACC A
NonTemplate Activator 3	5'- C GAC TCA CTA TA **G** *GGT CGA AGT TCA AGT CTG GT*
NonTemplate 3	5'- AC CAG ACT TGA ACT TCG ACC CTA TAG TGA GTC GTA TTA A CCA GAC ACG TAA G

Supplementary Table 8. Connector system for low yield design

**Connector system**	**Sequence**
Released Strand 1	5'- TGG TAT TGT CTG GT TAA TA
Non Released Strand 1	5'- ACC AGA CA ATA CCA ATC CGC
Released Strand 2	5'- CTT ACG TGT CTG GT TAA TA
Non Released Strand 2	5' – ACC AGA CA CGT AAG GAT GGC

Supplementary Table 9. Modified sequences for low yield design

**Modified strands**	**Sequence**
Released Strand 1	5'- TGG TAT TGT CTG GT TAA TA - (Cy3)

Non Released Strand 1	5'- (BHQ-1) - ACC AGA CA ATA CCA ATC CGC
Released Strand 2	5'- CTT ACG TGT CTG GT TAA TA - (Cy5)
Non Released Strand 2	5'- (BHQ-2)- ACC AGA CA CGT AAG GAT GGC

Supplementary Figures

Green system: Tile 1

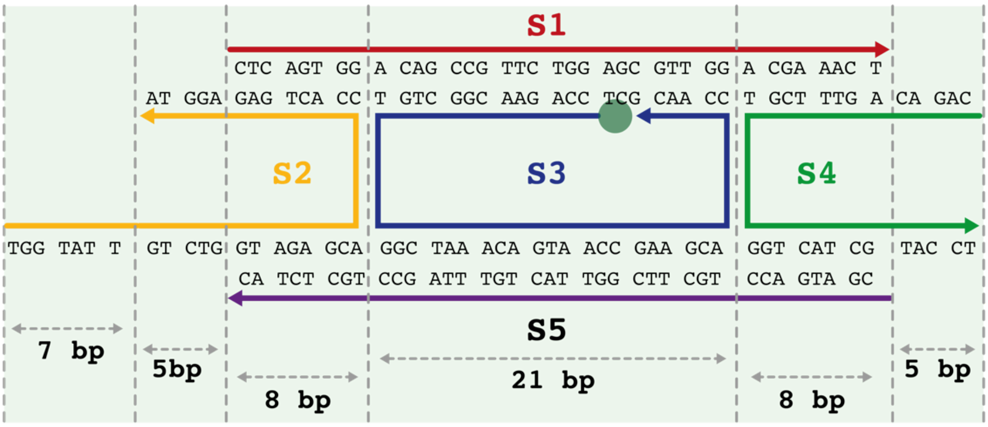

Supplementary Figure 1. *5-nucleotide sticky end green tile system. DNA tiles consist of 5 ssDNA (S1-S5). Here each strand is highlighted with a different color. The arrow indicates the 3' end.*

Red system: Tile 2

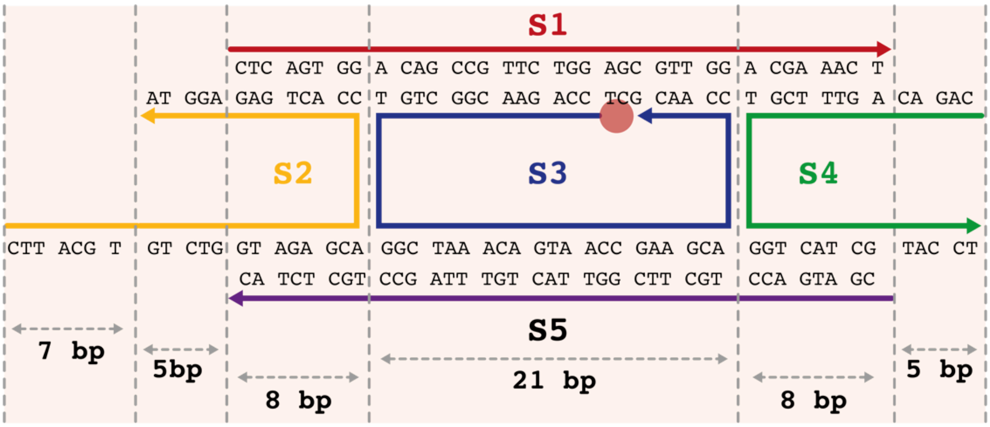

Supplementary Figure 2. *5-nucleotide sticky end red tile system. DNA tiles consist of 5 ssDNA (S1-S5). Here each strand is highlighted with a different color. The arrow indicates the 3' end.*

Blue system: Tile 3

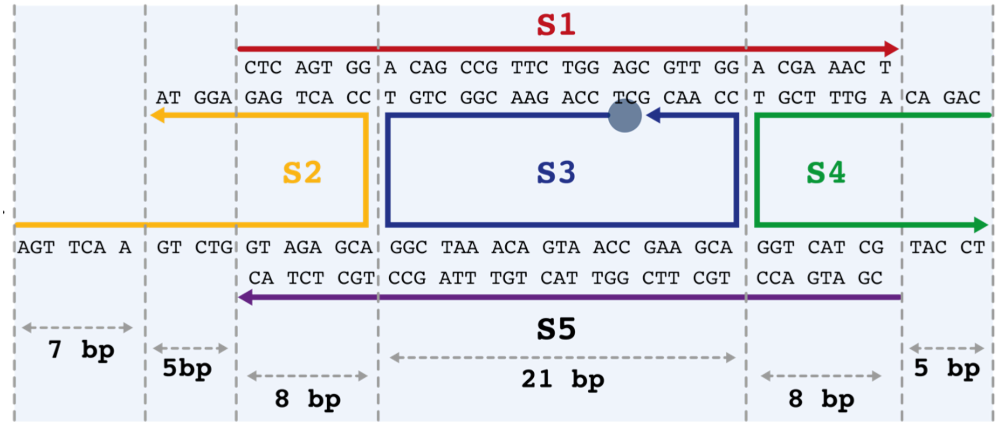

Supplementary Figure 3. *5-nucleotide sticky end blue tile system. DNA tiles consist of 5 ssDNA (S1-S5). Here each strand is highlighted with a different color. The arrow indicates the 3' end.*

High yield genes for DNA tile activation

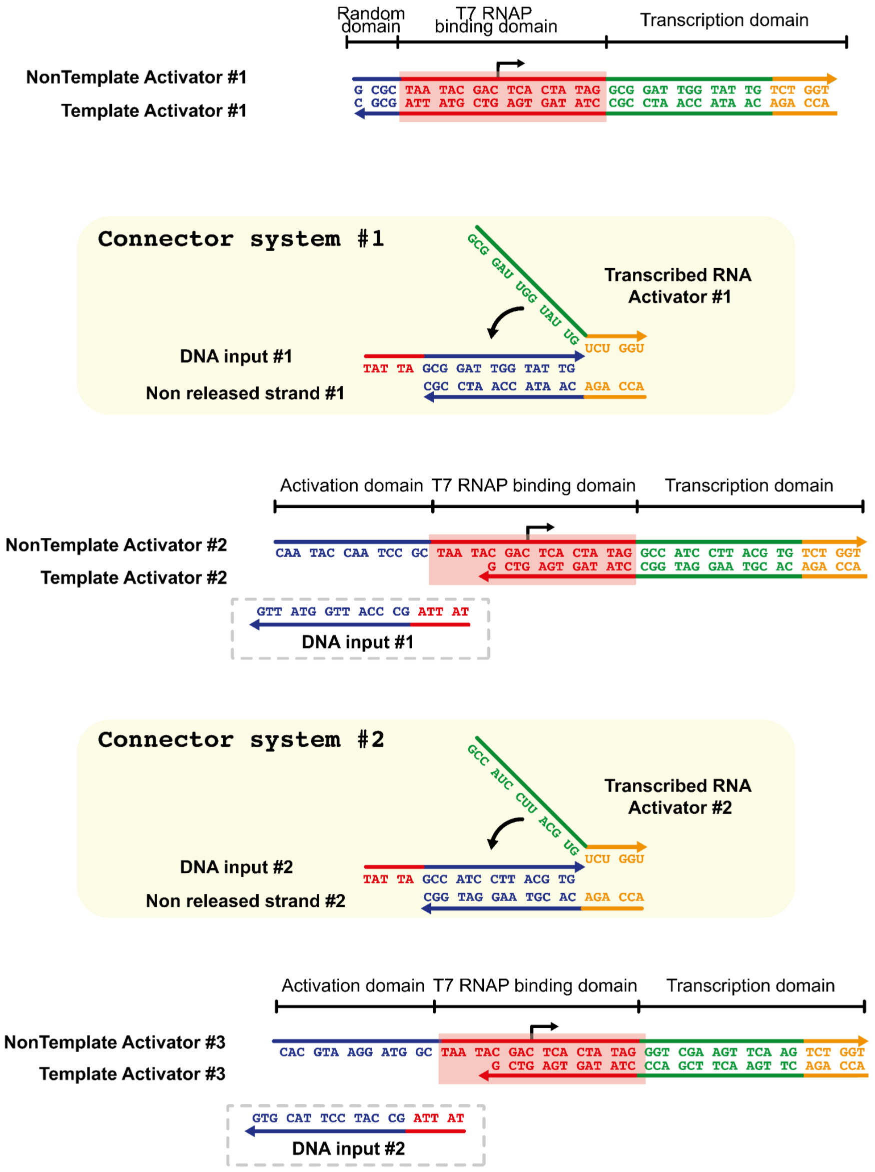

Supplementary Figure 4. *Schematic representation of how a cascade of different genes can be connected by a connector system. The second gene of the network is switched ON, when a DNA input is released from a connector complex. The gene is designed to be nicked on the side of the template, resulting in a high yield of RNA transcript that activates the corresponding tile to self-assemble into colored DNA nanotubes.*

Low yield genes for DNA tile activation

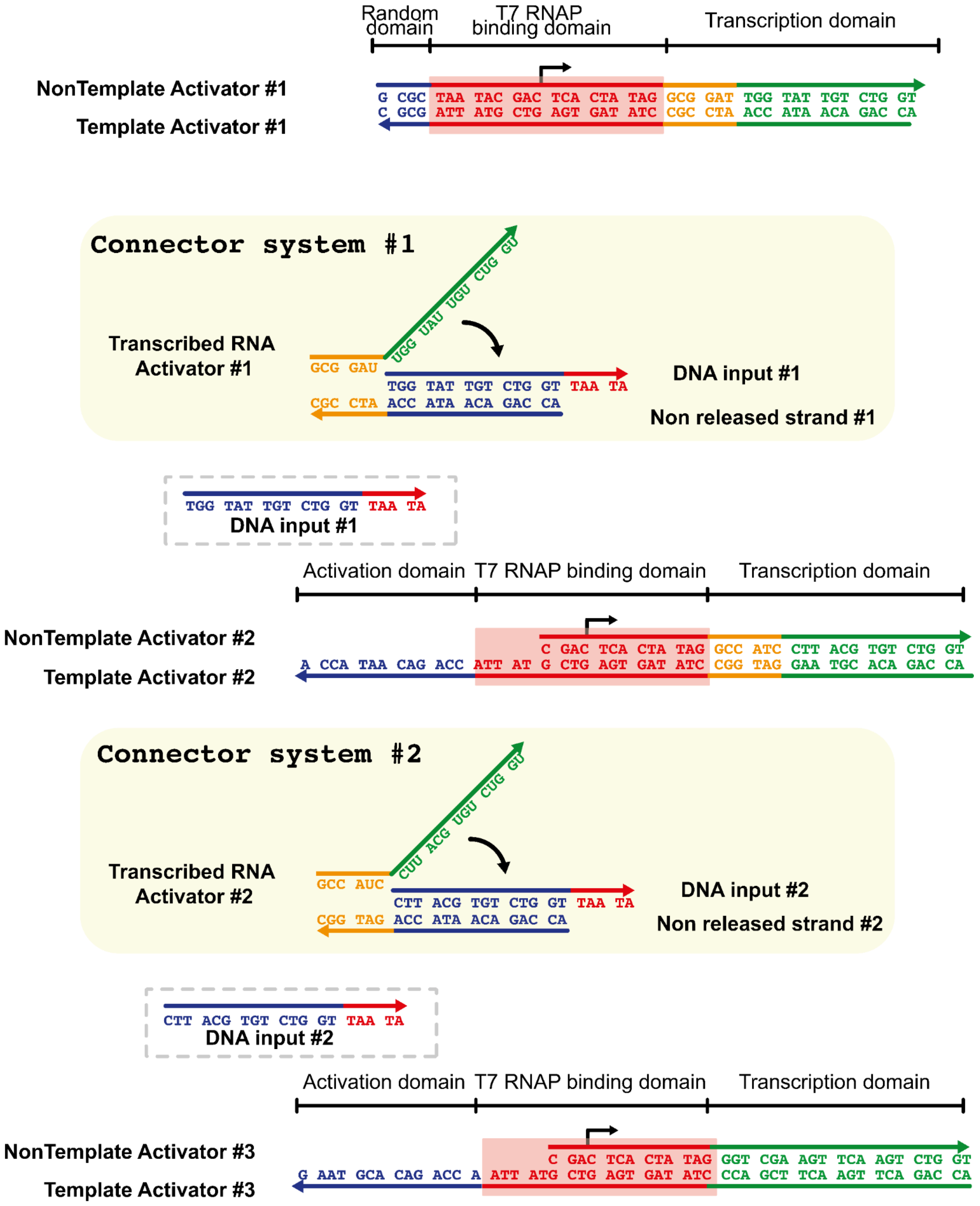

Supplementary Figure 5. *Schematic representation of how a cascade of different genes can be connected by a connector system. The second gene of the network is switched ON, when a DNA input is released from a connector complex. The gene is designed to be nicked on the non template side, resulting in a low yield of RNA transcript that activates the corresponding tile to self-assemble into colored DNA nanotubes.*

High yield genes for DNA tile inhibition

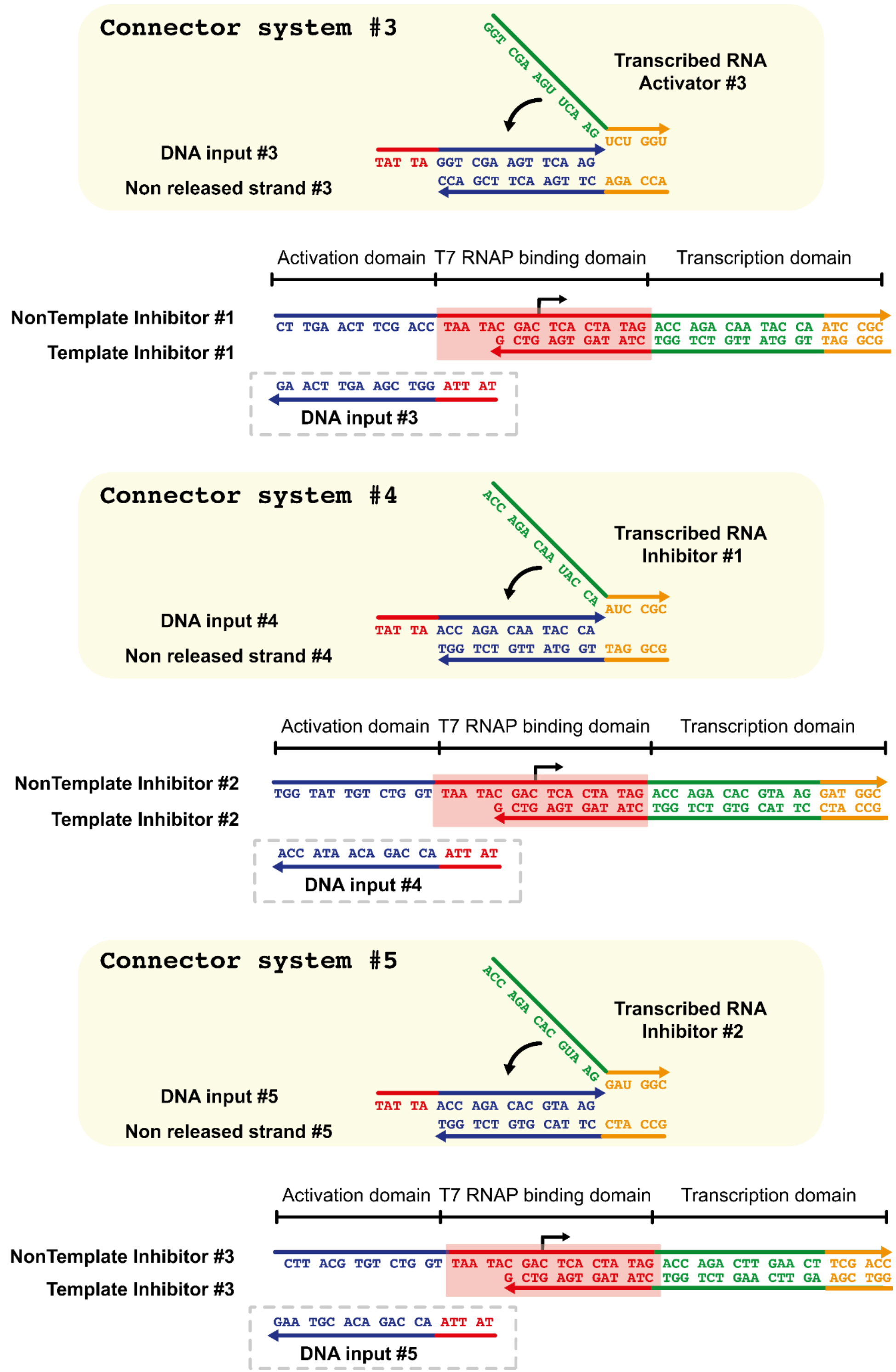

Supplementary Figure 6. *Schematic representation of how a cascade of different genes can be connected by a connector system. The gene is designed to be nicked on the template side, resulting in a high yield of RNA transcript that inhibits the corresponding tile to self-assemble into colored DNA nanotubes.*

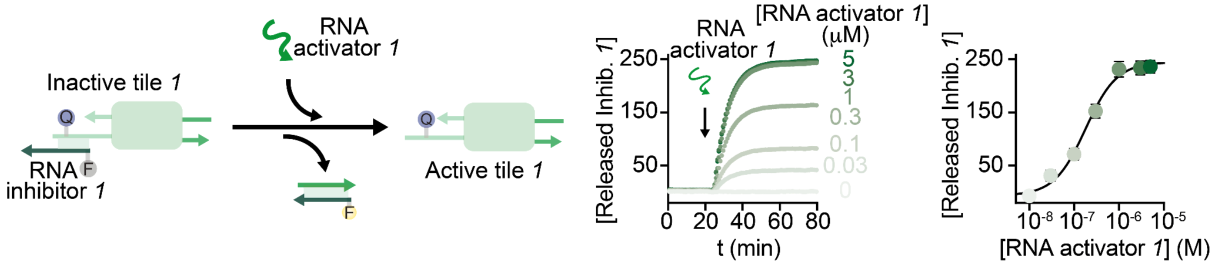

Supplementary Figure 7. Left. *Tile 1 activation using synthetic RNA activator 1.* ***Middle.*** *Released inhibitor 1 at different concentrations of synthetic RNA activator 1 in the presence of tile 1 (250 nM) and RNA inhibitor 1 (1 µM).* ***Right.*** *End point values vs RNA activator 1 concentration. Experiments were performed at 30° C in 1X transcription buffer, 10 mM NTPs, pH 8.0 in a 100 µL cuvette. Experimental values are averages of three separate measurements and error bars reflect standard deviations.*

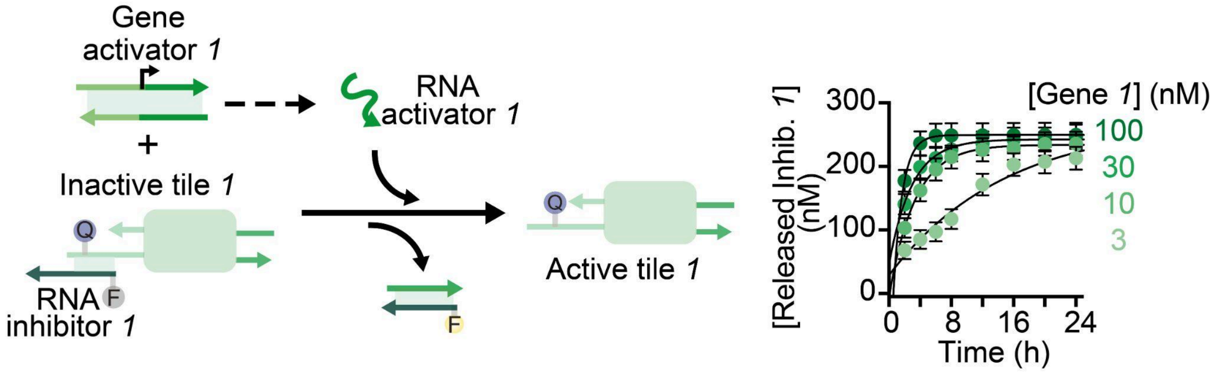

Supplementary Figure 8. Left. *Tile 1 (250 nM) activation using gene activator 1 (100 nM).* ***Right.*** *Kinetic traces showing the release of the RNA inhibitor over time at different concentrations of gene activator 1. Experiments were performed at 30° C in 1X transcription buffer, 10 mM NTPs, T7 RNP 4 U/µL, pH 8.0 in a 100 µL cuvette. Experimental values are averages of three separate measurements and error bars reflect standard deviations.*

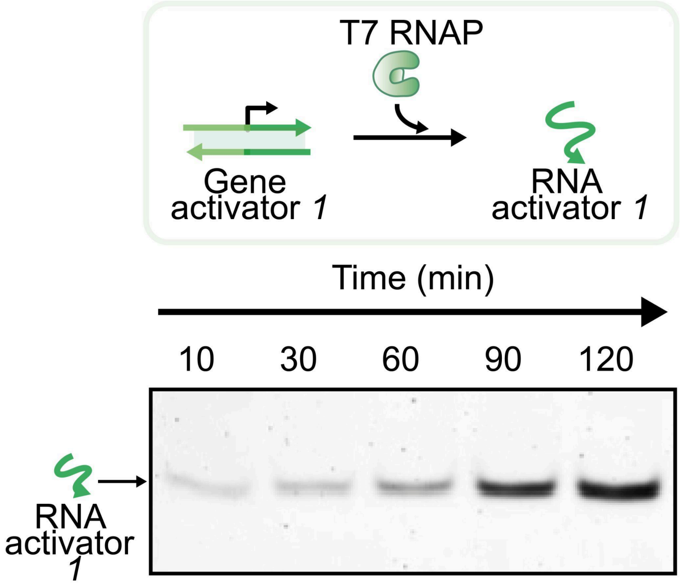

Supplementary Figure 9. *Native PAGE gel (15% polyacrylamide) showing production of RNA activator 1 over time. Each solution containing gene activator 1 (100 nM) was prepared in a 1X transcription buffer, NTPs (10 mM), T7 RNAP (4 U/μL). Gel was run at 25°C (110 V) for 2h 30 min in 1x TBE buffer, pH 8.0, stained with 1x SYBR-Gold, and imaged using the Gel Doc XR system (Bio-Rad).*

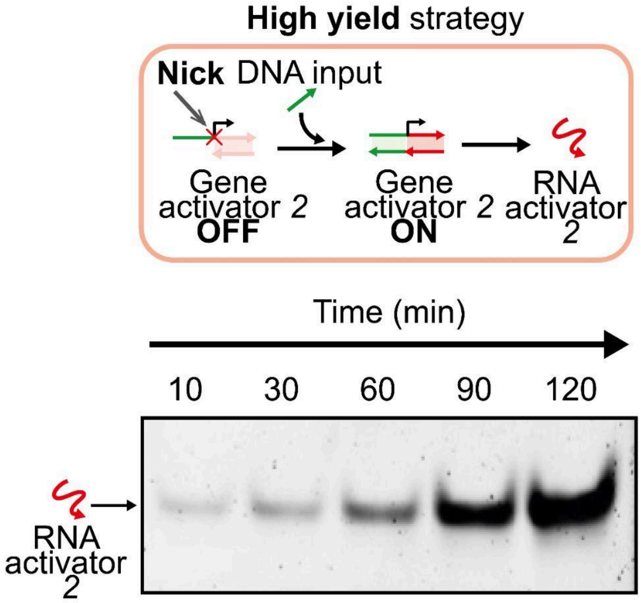

Supplementary Figure 10. *Native PAGE gel (15% polyacrylamide) showing production of RNA activator 2 from a high yield gene over time. Each solution containing high yield gene 2 (100 nM), and DNA input (100 nM) was prepared in a 1X transcription buffer, NTPs (10 mM), T7 RNAP (4 U/μL). Gel was run at 25°C (110 V) for 2h 30 min in 1x TBE buffer, pH 8.0, stained with 1x SYBR-Gold, and imaged using the Gel Doc XR system (Bio-Rad).*

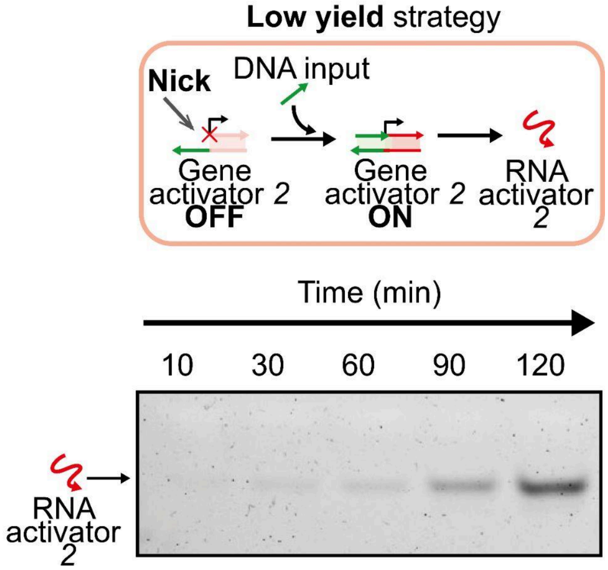

Supplementary Figure 11. *Native PAGE gel (15% polyacrylamide) showing production of RNA activator 2 from a low yield gene over time. Each solution containing low yield gene 2 (100 nM), and DNA input (100 nM) was prepared in a 1X transcription buffer, NTPs (10 mM), T7 RNAP (4 U/µL). Gel was run at 25°C (110 V) for 2h 30 min in 1x TBE buffer, pH 8.0, stained with 1x SYBR-Gold, and imaged using the Gel Doc XR system (Bio-Rad).*

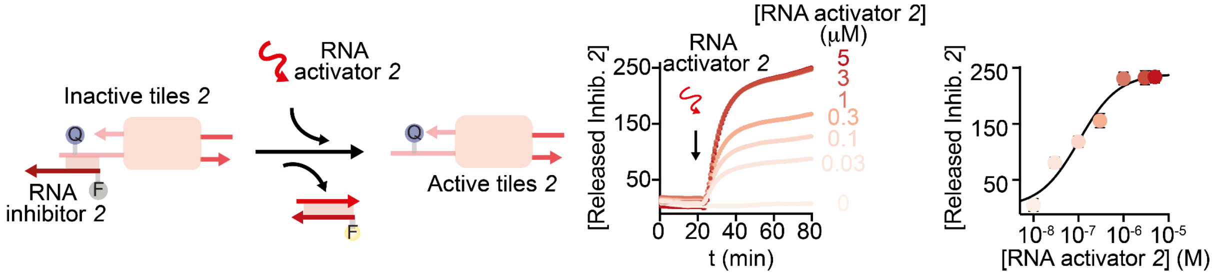

Supplementary Figure 12. Left. *Tile 2 activation using synthetic RNA activator 2.* ***Middle.*** *Released inhibitor 2 at different concentrations of synthetic RNA activator 2 in the presence of tile 2 (250 nM) and RNA inhibitor 2 (1 µM).* ***Right.*** *End point values vs RNA activator 2 concentration. Experiments were performed at 30° C, in 1X transcription buffer, 10 mM NTPs, pH 8.0 in a 100 µL cuvette. Experimental values are averages of three separate measurements (n=3) and error bars reflect standard deviations .*

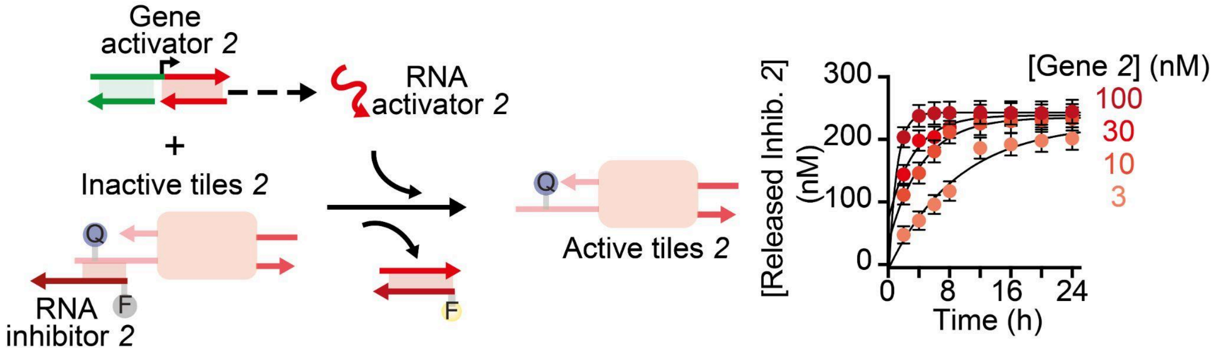

Supplementary Figure 13. Left. *Tile 2 (250 nM) activation using gene activator 2 (100 nM).* ***Right.*** *Kinetic traces showing the release of the RNA inhibitor 2 over time at different concentrations of high yield gene activator 2. Experiments were performed at 30° C in 1X transcription buffer, 10 mM NTPs, T7 RNP 4 U/ uL, pH 8.0 in a 100 µL cuvette. Experimental values are averages of three separate measurements (n=3) and error bars reflect standard deviations.*

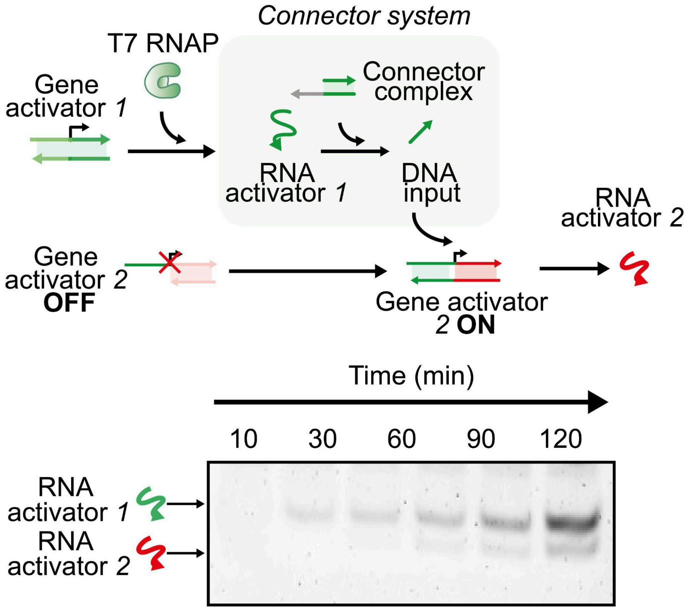

Supplementary Figure 14. Top. *Schematic representation of a simplified cascade network of two interconnected synthetic genes (1 and 2).* ***Bottom.*** *Native PAGE gel (15% polyacrylamide) showing production of RNA activator 1 and 2 over time. We achieve a tunable delay in the production of RNA activator 2 by confirming the gene is not switched to ON until its promoter is completed. Each solution containing gene activator 1 and 2 (100 nM), and connector complex (300 nM) was prepared in a 1X transcription buffer, NTPs (10 mM), T7 RNAP (4 U/µL). Gel was run at 25°C (110 V) for 2h 30 min in 1x TBE buffer, pH 8.0, stained with 1x SYBR-Gold, and imaged using the Gel Doc XR system (Bio-Rad).*

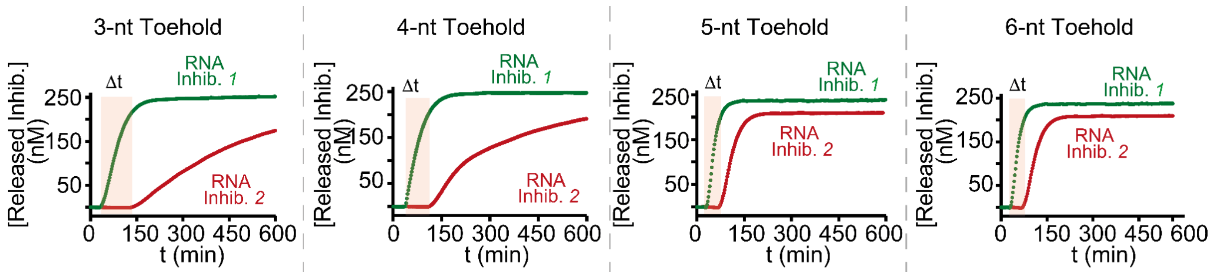

Supplementary Figure 15. *Fluorescence kinetic traces showing the release of RNA inhibitor strands 1 and 2 from their corresponding tiles triggered by the presence of transcribed RNA activator strands 1 and 2 and the connector complex at different toehold length (from 3 to 6-nt). Each solution containing tile 1 and 2 (250 nM), RNA inhibitors 1 and 2 (1 µM each), gene 1 and high yield gene 2 (100 nM each), and the connector complex (300 nM) was prepared in a 1X transcription buffer, NTPs (10 mM), T7 RNAP (4 U/µL).*

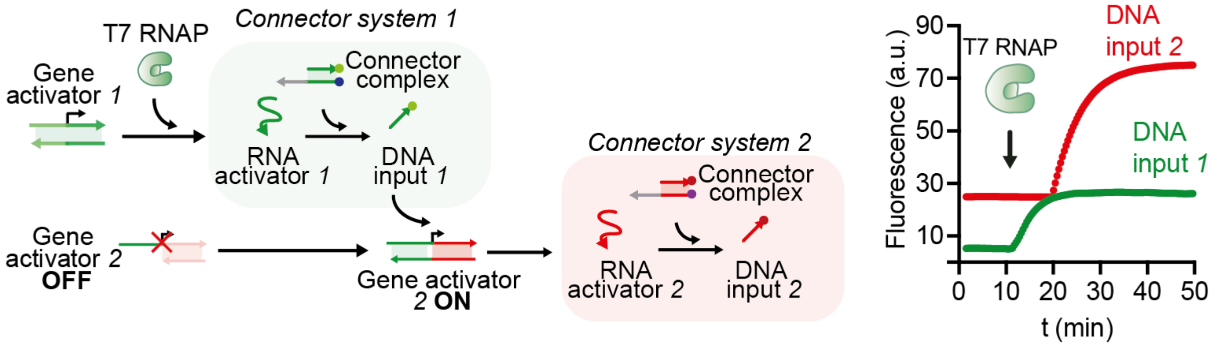

Supplementary Figure 16. Left. *Schematic representation of the displacement of a DNA input from a connector complex upon RNA transcription.* ***Right.*** *Kinetic traces showing release of DNA input from fluorophore/quencher labeled connector complexes at fixed concentrations of gene 1 and high yield gene 2 (100 nM each). Each solution containing tile 1 and 2 (250 nM), RNA inhibitors (1 µM), gene activators 1 and 2 (100 nM), and the connector complexes (300 nM) was prepared in a 1X transcription buffer, NTPs (10 mM), T7 RNAP (4 U/µL).*

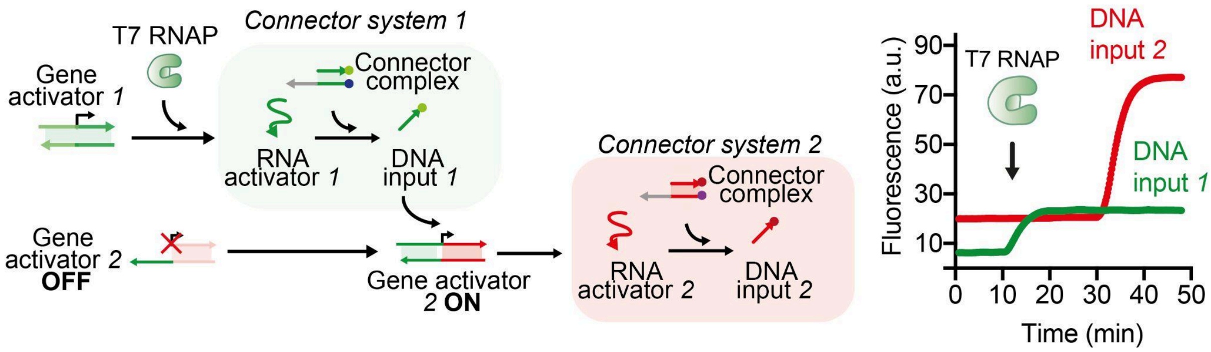

Supplementary Figure 17. *Schematic representation of the displacement of a DNA input from a connector complex upon RNA transcription.* ***Right.*** *Kinetic traces showing release of DNA input from fluorophore/quencher labeled connector complexes at fixed concentrations of gene 1 and low yield yield gene 2 (100 nM each). Each solution containing tile 1 and 2 (250 nM), RNA inhibitors (1 µM), gene activators 1 and 2 (100 nM), and the connector*

complexes (300 nM) was prepared in a 1X transcription buffer, NTPs (10 mM), T7 RNAP (4 U/µL).

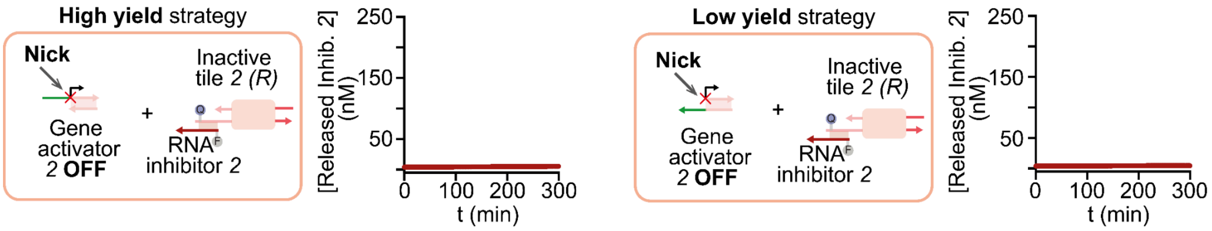

Supplementary Figure 18. *Kinetic traces showing that negligible transcription occurs when both the high yield and low yield genes are not complemented with their corresponding DNA inputs under in vitro transcription conditions. The release of RNA inhibitor 2 from the corresponding tiles (250 nM) does not occur at fixed concentrations of gene 1 and high yield gene 2 (100 nM each), and a fixed concentration of T7 RNAP (4 U/µL). To easily follow the release of the inhibitors after production of the RNA activator, we modified the RNA inhibitor and the interacting tile strand with an appropriate fluorophore and quencher pair (Cy5/BHQ2).*

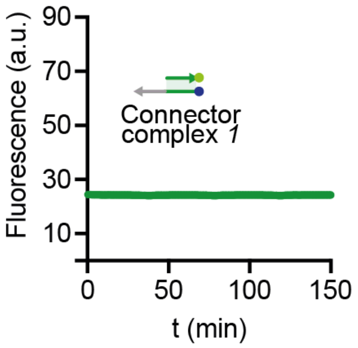

Supplementary Figure 19. *Kinetic trace showing the stability of the fluorophore/quencher labeled connector complex (300 nM) under in vitro transcription conditions (in 1X transcription buffer, NTPs (10 mM), T7 RNAP (4 U/µL)).*

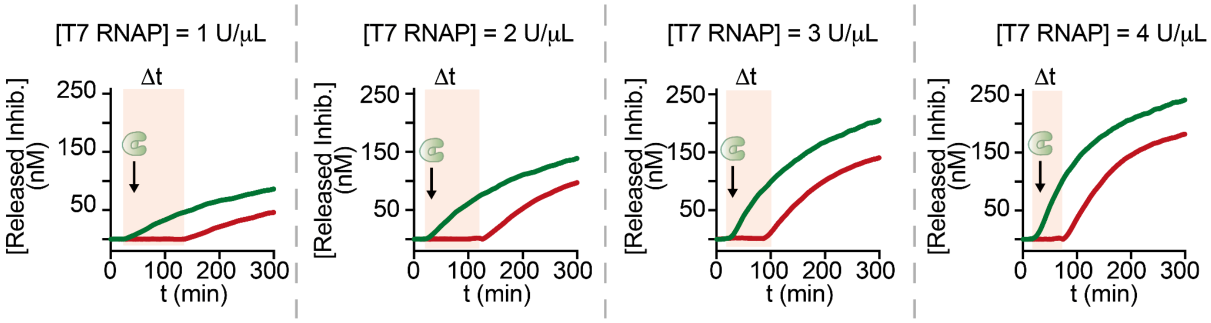

Supplementary Figure 20. *Kinetic traces showing the release of RNA inhibitors (1 and 2) from the corresponding tiles at fixed concentrations of gene 1 and high yield gene 2 (100 nM each), connector complex (300 nM), and various concentrations of T7 RNAP (from 1 to 4 U/µL). To easily follow the release of the inhibitors after production of the RNA activator, we modified the RNA inhibitor and interacting tile strand with an appropriate fluorophore and quencher pair (1, Cy3/BHQ1 and 2, Cy5/BHQ2).*

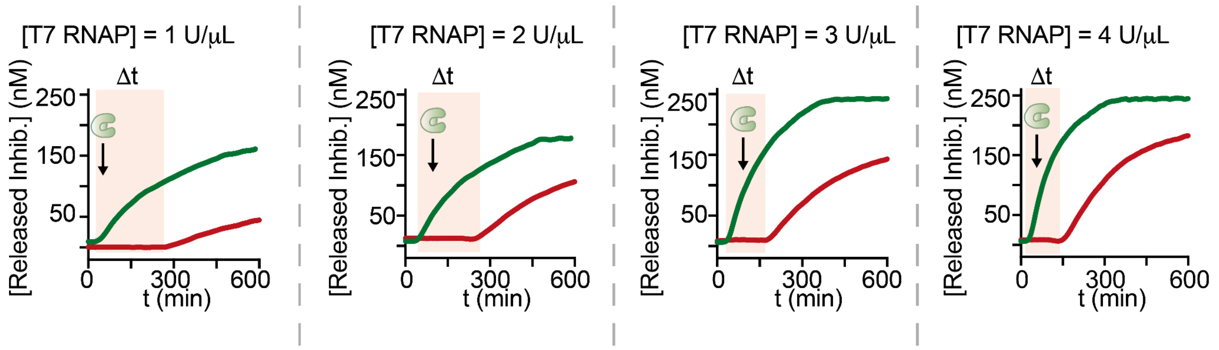

Supplementary Figure 21. *Kinetic traces showing the release of RNA inhibitors (1 and 2) from the corresponding tiles at fixed concentrations of gene 1 and low yield gene 2 (100 nM each), connector complex (300 nM), and various concentrations of T7 RNAP (from 1 to 4 U/μL). To easily follow the release of the inhibitors after production of the RNA activator, we modified the RNA inhibitor and interacting tile strand with an appropriate fluorophore and quencher pair (1, Cy3/BHQ1 and 2, Cy5/BHQ2).*

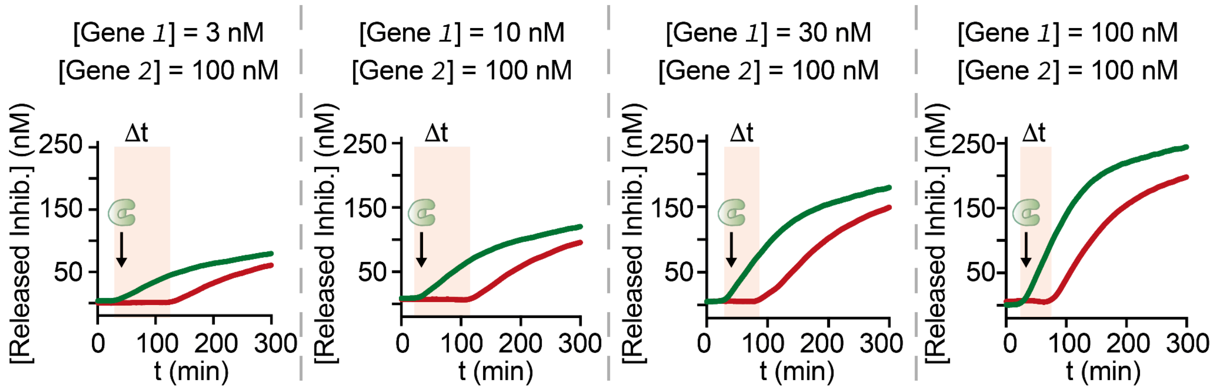

Supplementary Figure 22. *Kinetic traces showing the release of RNA inhibitors (1 and 2) from the corresponding tiles at different concentrations of gene 1 (from 3 to 100 nM), fixed concentration of high yield gene 2 (100 nM), connector complex (300 nM), and T7 RNAP (4 U/uL). To easily follow the release of the inhibitors after the production of the RNA activator, we modified the RNA inhibitor and the interacting tile strand with a fluorophore and quencher pair (1, Cy3/BHQ1 and 2, Cy5/BHQ2).*

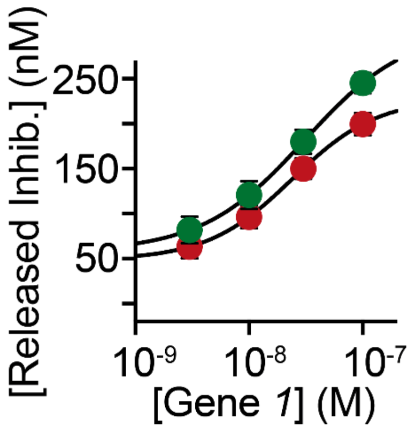

Supplementary Figure 23. *Release of RNA inhibitors 1 (green) and 2 (red) from the corresponding DNA tiles after 300 min as a function of gene 1 concentration. Experimental*

values are averages of three separate measurements (n=3) and error bars reflect standard deviations.

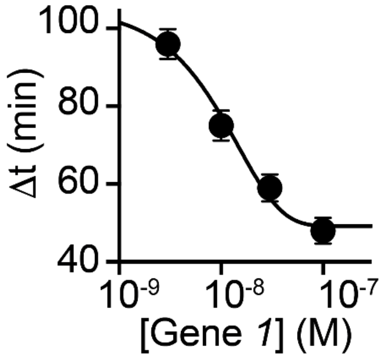

Supplementary Figure 24. *Delay time (Δt) of tile 2 activation as a function of gene 1 concentration. Experimental values are averages of three separate measurements (n=3) and error bars reflect standard deviations.*

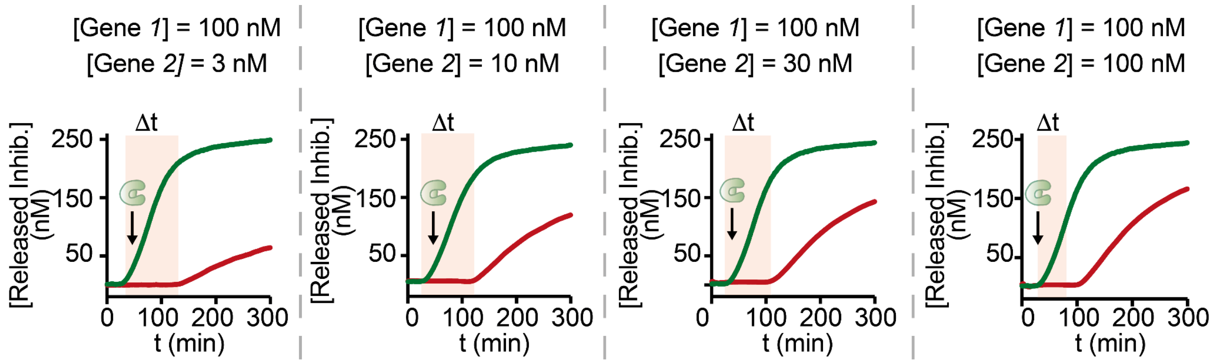

Supplementary Figure 25. *Kinetic traces showing the release of RNA inhibitors (1 and 2) from the corresponding tiles at different concentrations of high yield gene 2 (from 3 to 100 nM), fixed concentration of gene 1 (100 nM), connector complex (300 nM), and T7 RNAP (4 U/μL). To easily follow the release of the inhibitors after the production of the RNA activator, we modified the RNA inhibitor and the interacting tile strand with a fluorophore and quencher pair (1, Cy3/BHQ1 and 2, Cy5/BHQ2).*

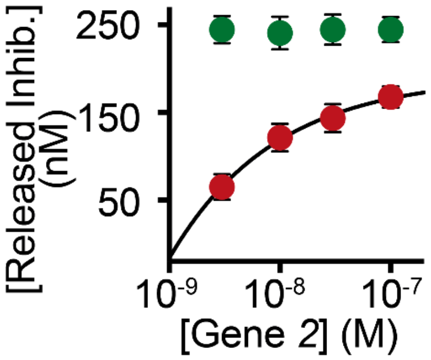

Supplementary Figure 26. *Release of RNA inhibitors 1 (green) and 2 (red) from the corresponding DNA tiles after 300 min as a function of gene 2 concentration. Experimental values are averages of three separate measurements (n=3) and error bars reflect standard deviations.*

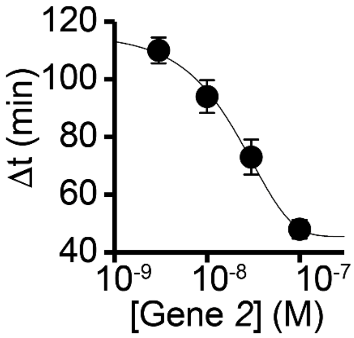

Supplementary Figure 27. *Delay time (Δt) of tile 2 activation as a function of high yield gene 2 concentration. Experimental values are averages of three separate measurements (n=3) and error bars reflect standard deviations.*

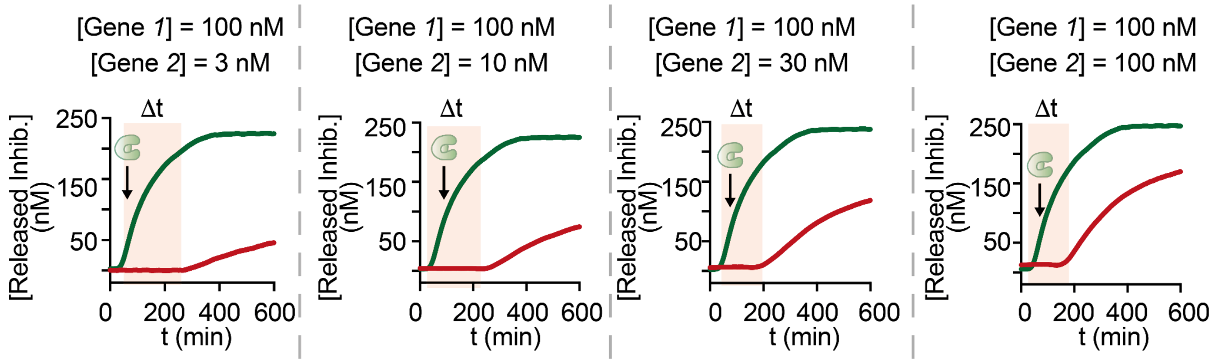

Supplementary Figure 28. *Kinetic traces showing the release of RNA inhibitors (1, green and 2, red) from the corresponding tiles at different concentrations of low yield gene 2 (from 3 to 100 nM), fixed concentration of gene 1 (100 nM), connector complex (300 nM), and T7 RNAP (4 U/μL). To easily follow the release of the inhibitors after the production of the RNA activator, we modified the RNA inhibitor and the interacting tile strand with a fluorophore and quencher pair (1, Cy3/BHQ1 and 2, Cy5/BHQ2).*

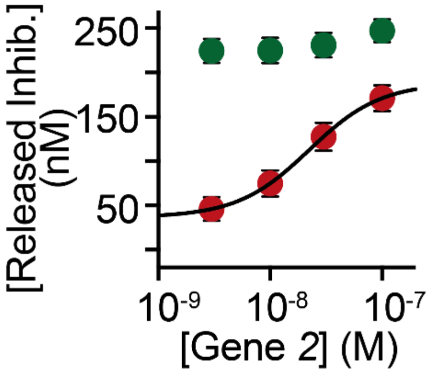

Supplementary Figure 29. *Release of RNA inhibitors 1 (green) and 2 (red) from the corresponding DNA tiles after 600 min as a function of low yield gene 2 concentration. Experimental values are averages of three separate measurements (n=3) and error bars reflect standard deviations.*

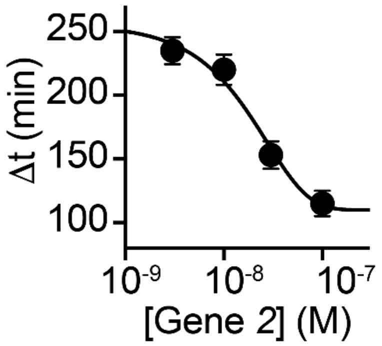

Supplementary Figure 30. *Delay time (Δt) of tile 2 activation as a function of low yield gene 2 concentration. Experimental values are averages of three separate measurements (n=3) and error bars reflect standard deviations.*

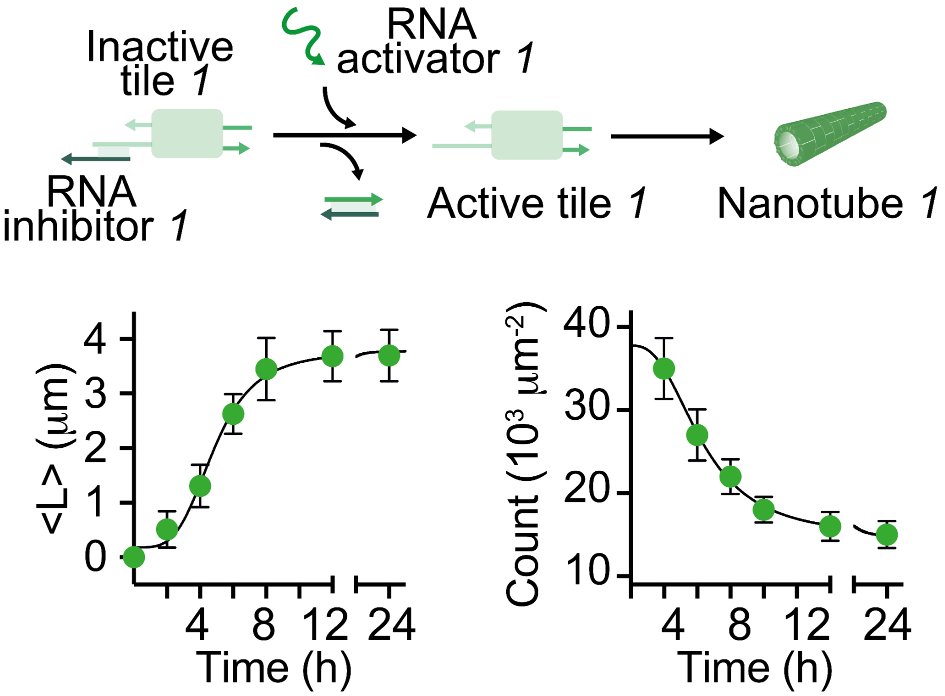

Supplementary Figure 31. Top. *Schematic representation of a control experiment in which the inactivation and activation of the tiles and thus the growth of the nanotubes is mediated by the presence of synthetic RNA strands (i.e. inhibitor or activator).* ***Bottom left.*** *Kinetic*

traces of nanotube length measured from fluorescence microscopy images.<L>indicates the mean nanotube length of nanotubes. ***Bottom right.*** *Count (number of structures per 100 μm²) of assembled green nanotubes measured from fluorescence microscopy images. Experiments shown in this Fig. were performed in 1X TXN buffer (5X contains: 200 mM Tris-HCl, 30 mM $MgCl_2$, 50 mM DTT, 50 mM NaCl, and 10 mM spermidine), pH 8.0, 30 °C. [Tile G, 1] = 250 nM; [RNA inhibitor 1] = 1 μM; [RNA activator 1] = 3 μM. Experimental values are averages of three separate measurements (n=3) and error bars reflect standard deviations.*

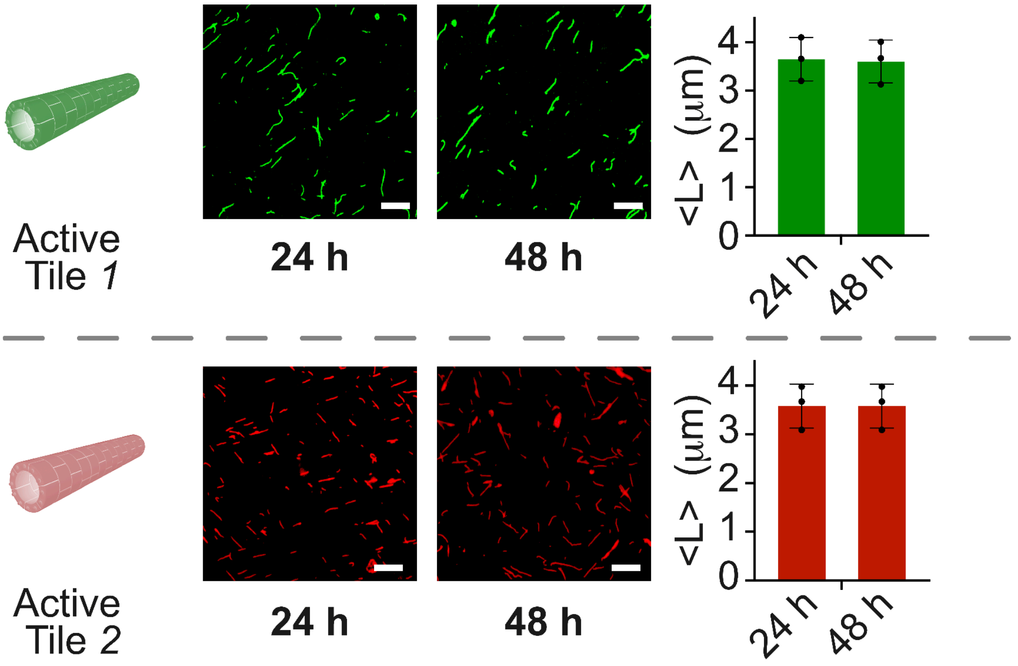

Supplementary Figure 32. *Control experiments for testing nanotubes stability under in vitro transcription conditions imaged at 24, and 48 h after the annealing step. Nanotube length measured from fluorescence microscopy images.<L>indicates the mean nanotube length of nanotubes. Experiments shown in this Fig. were performed in 1X TXN buffer (5X contains: 200 mM Tris-HCl, 30 mM $MgCl_2$, 50 mM DTT, 50 mM NaCl, and 10 mM spermidine), pH 8.0, 30 °C. [Tile G, 1] = [Tile R, 2] 250 nM. Scale bars are 2.5 μm. Experimental values are averages of three separate measurements (n=3) and error bars represent standard deviation and the centre of the error bars represents the mean.*

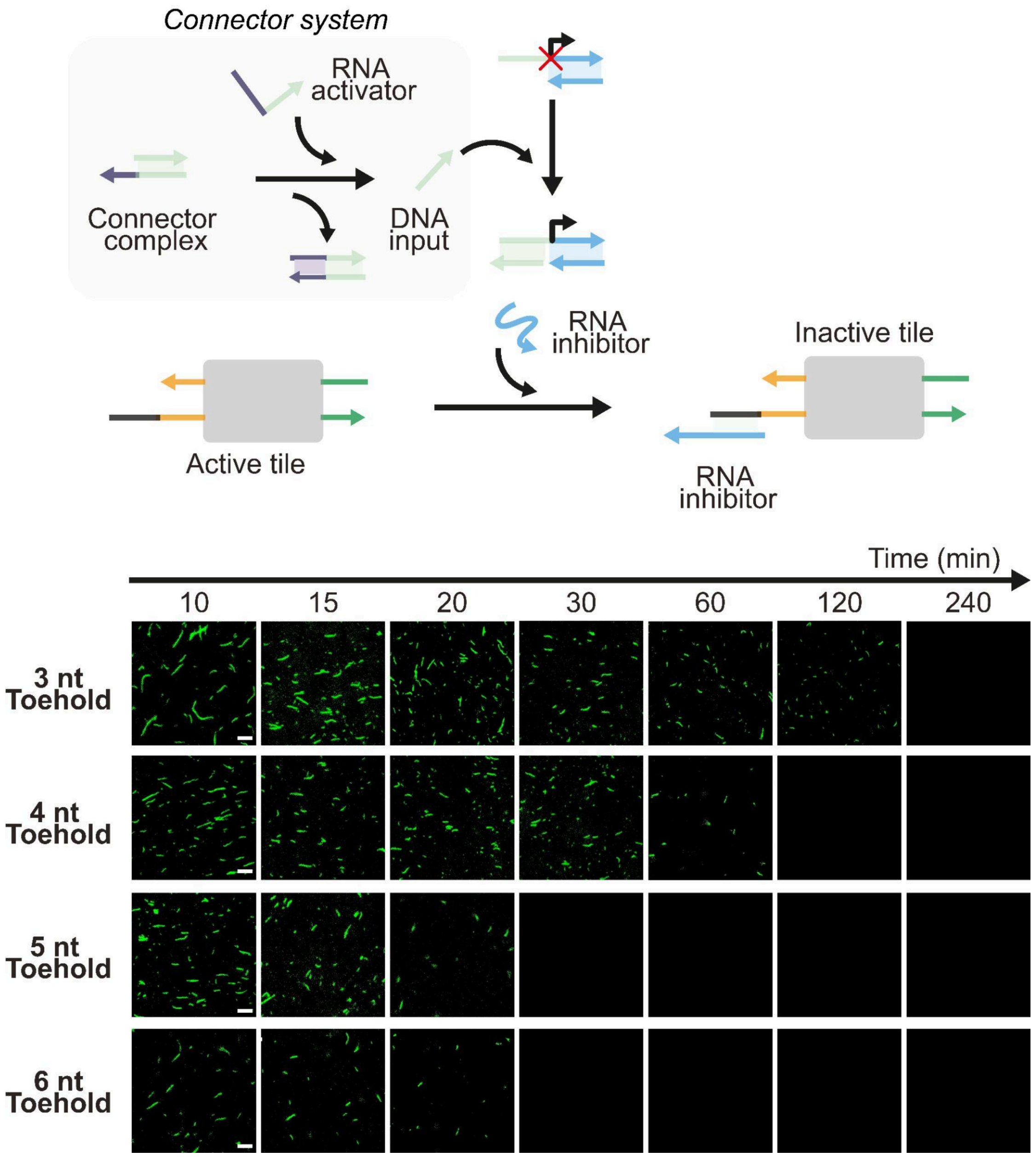

Supplementary Figure 33. Top. *Schematic representation of tile inactivation mediated by the presence of a transcribed RNA inhibitor.* ***Bottom.*** *Kinetics of disassembly of tile G can be modulated by the length of the toehold of one of the strands forming the connector complex. Experiments were performed in the presence of active tiles G (250 nM), connector complex (300 nM), RNA activator (1 µM), inhibitor gene (100 nM). Fluorescence images scale bar, 2.5 µm*

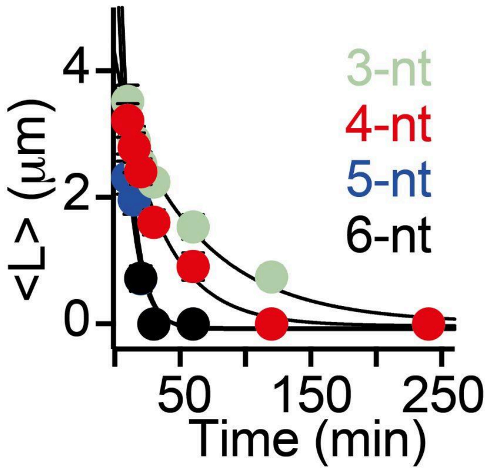

Supplementary Figure 34. *Nanotube mean length at different lengths of the toehold portion (3-nt, green; 4-nt, red; 5-nt, blue; 6-nt, black) of one of the strands of the connector complex vs time (see SI 23). <L> indicates the mean nanotube length of nanotubes. Experimental values are averages of three separate measurements (n=3) and error bars represent standard deviation.*

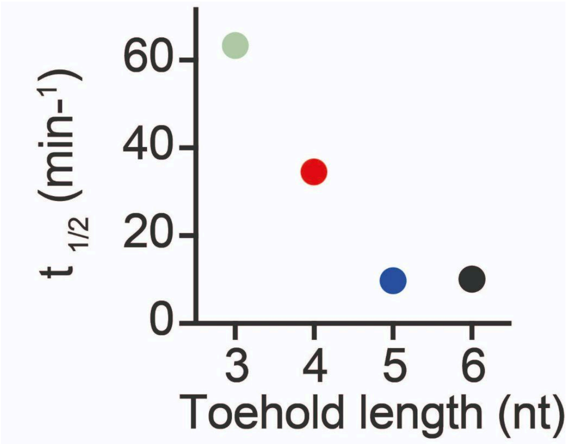

Supplementary Figure 35. Reaction half-life ($t_{1/2}$) of tile inhibition as a function of toehold length (from 3 to 6-nt).

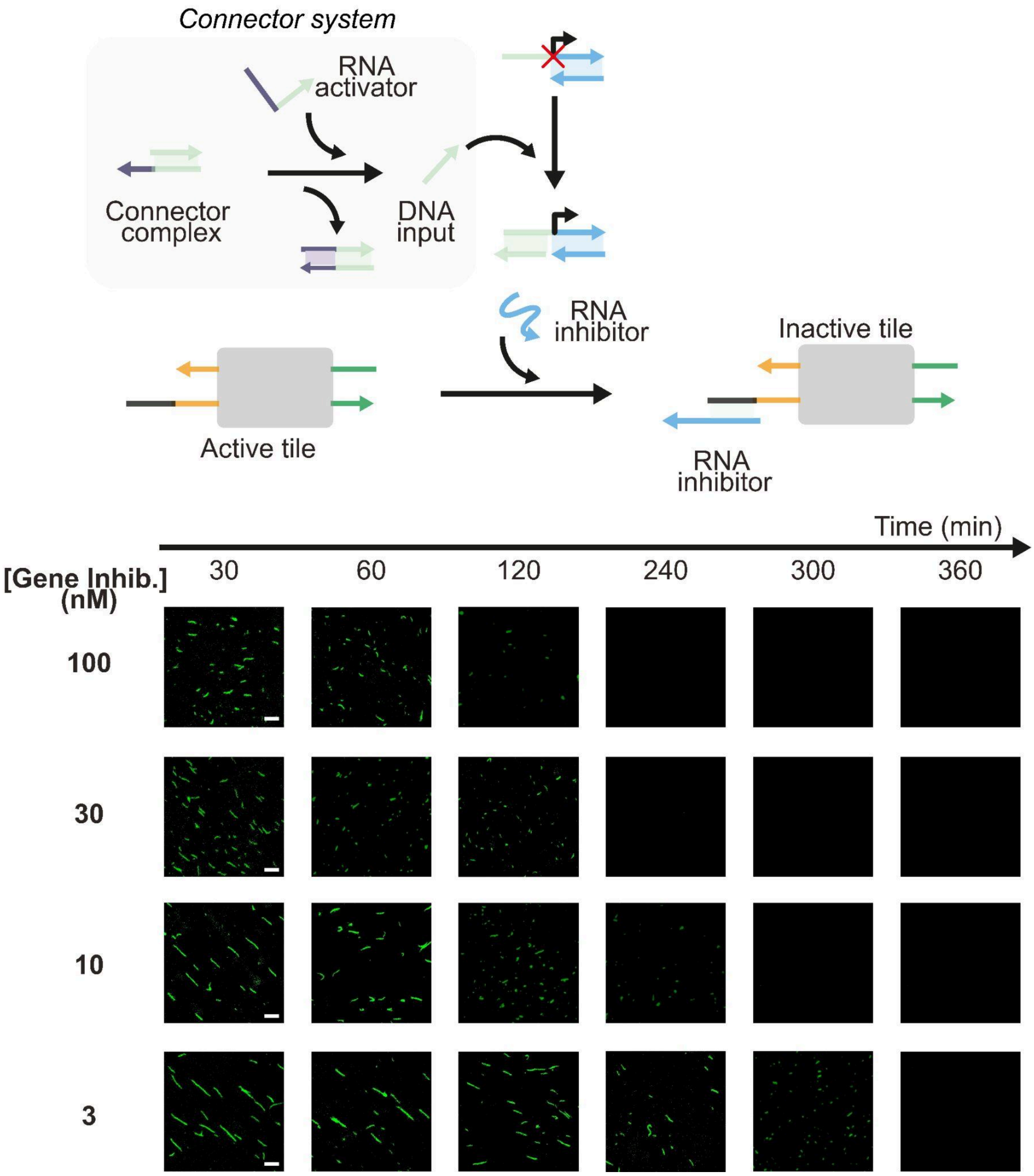

Supplementary Figure 36. Top. *Schematic representation of tile inactivation by the presence of a transcribed RNA inhibitor.* ***Bottom.*** *The kinetics of degradation of the active G tiles can be modulated by the concentration of the gene producing the RNA inhibitor by using 3-nt toehold of one of the strands forming the connector complex. Experiments were performed in the presence of active tiles G (250 nM), connector complex (300 nM), RNA activator (1 µM), inhibitor gene (3-100 nM). Fluorescence images scale bar, 2.5 µm.*

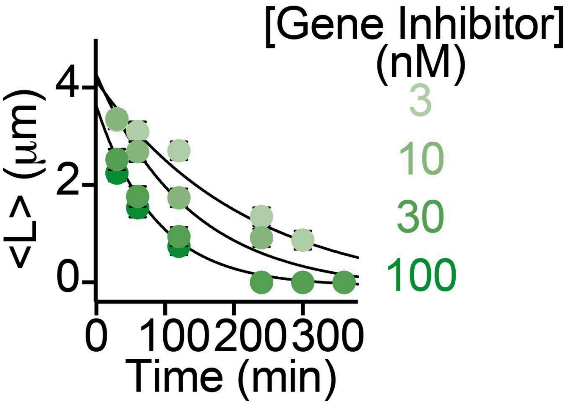

Supplementary Figure 37. *Kinetic traces of nanotubes mean length. The rate of tile disassembly depends on the concentration of the gene transcribing the RNA inhibitor strand (from 3 to 100 nM; light to dark green). <L> indicates the mean nanotube length of nanotubes. Experimental values are averages of three separate measurements (n=3) and error bars represent standard deviation.*

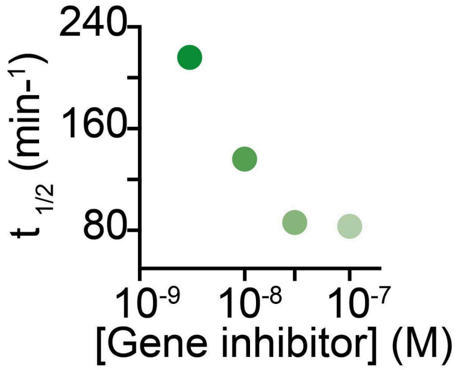

Supplementary Figure 38. *Reaction half-life ($t_{1/2}$) of tile inhibition as a function of gene inhibitor concentration.*

Supplementary Note 1: Data fitting

- The black curves reported in Fig. 2 d,i,m and Fig. 4 c,d,g,h,j,k,n,q of the manuscript were fitted using the following four parameter logistic equation (1) in Prism 9:

$$Y = Min + (Max - Min)/(1 + 10^{((LogEC50 - X)*HillSlope)}) \quad (1)$$

where:

EC50 is the concentration of agonists that gives a response halfway between Min and Max.

HillSlope describes the steepness of the family of curves.

Max and **Min** are plateaus of the curves in the units of the Y axis.

- The black curves in Fig. 2 f,k,o were fitted using the following one phase decay exponential equation (2) in Prism 9:

$$Y = (Y0 - Plateau) * exp(- K * X) + Plateau \quad (2)$$

where:

Y0 is the Y value when X (time) is zero. It is expressed in the same units as Y,
Plateau is the Y value at infinite times, expressed in the same units as Y.
K is the rate constant, expressed in reciprocal of the X axis time units. If X is in minutes, then K is expressed in inverse minutes.

Supplementary Note 2: Modeling

We modeled tile inhibition and activation through the following equivalent chemical reactions. Here species T represents active tiles, T^* represents inactive tiles, I is the RNA inhibitor, and R is the RNA activator:

$$T + I \xrightarrow{k_{TI}} T^*$$
$$T^* + R \xrightarrow{k_{TR}} T + IR$$
$$I + R \xrightarrow{k_{IR}} IR$$

We assume conservation of mass: $T^{tot} = T + T^*$, $I^{tot} = I + T^* + RI$.

To model *in situ* production of activator R through *in vitro* transcription through a gene G and RNA polymerase $RNAP$, we used the following reactions:

$$G + RNAP \xrightarrow{k^+} GRp \xrightarrow{k^-} G + RNAP$$

$$GRp \xrightarrow{k_{cat}} G + RNAP + R$$

When simulating two interconnected genes and tile systems (Fig. 2 of the manuscript), we used the reactions above, plus reactions modeling the release of DNA activators A_1 from connector complex S_1, and activation of gene G_2 through the following reactions:

$$R_1 + S_1 \xrightarrow{k_{RS}} A_2 + S_1^*$$
$$A_2 + G_2^* \xrightarrow{k_{AG}} G_2$$

where S_1^* and G_2* are respectively released connector complex and inactive gene. We assume mass conservation $S_1^{tot} = S_1 + S_1^*$ and $A_2^{tot} = S_1^{tot} = S_1 + A_2 + G_2$

The two independent sets of tiles, inhibitors, activators, connectors and genes were modeled through distinct species T_1, I_1, R_1, S_1, G_1 and T_2, I_2, R_2, G_2, assuming mass conservation for tiles, inhibitors, and genes. Tile inhibition/activation reactions were assumed to be the same for each subsystem, and rate constants were also assumed to be the consistent.

We used the reactions above to generate systems of ODEs, which were numerically integrated using MATLAB. Simulations including *in vitro* transcription assume zero initial amount of activator RNA. Other initial conditions are consistent with the experimental settings captured by the simulation. Total concentrations of templates, inhibitors, and genes were set to be consistent with experiments. We compared simulations with tile 1 activation experiments only (as tile 2 experiments yield similar results), reported in Supplementary Fig.s 30, 31, and 32. The simulations of the interconnected genes and tiles were used to generate the estimates for half-time and delay reported in Fig. 3 of the manuscript.

Reaction rate parameters were estimated from values provided in the literature[5–8]: $k_{TR} = 5 \cdot 10^5/M/s$, $k_{TI} = 1 \cdot 10^2/M/s$, $k_{IR} = 3 \cdot 10^2/M/s$, $k_{RS} = 4 \cdot 10^4/M/s$, and $k_{AG} = 3 \cdot 10^3/M/s$. Low values for the hybridization of free T and I, and free R and I are based on the experimental control experiments in Supplementary Fig. 4, reported also in Supplementary Fig. 30 for comparison with the model. To reproduce experiments in Fig. 3d and e of the manuscript, in which the toehold length of the connector complex varies, we used the release rate constant k_{RS} reported above to simulate the 6 nt toehold; for shorter toeholds, we scaled this constant by a factor 0.7, 0.03, 0.002 respectively for 5, 4, and 3 nt toeholds.

For in vitro transcription, we used the following parameters when simulating the high yield genes: $k^+ = 1 \cdot 10^6/M/s$, $k^- = 1 \cdot 10^{-3}/s$, and $k_{cat} = 1 \cdot 10^{-3}/s$. For the low yield genes, we assumed $k^+ = 1 \cdot 10^5/M/s$ (slower binding when compared to the high yield case), $k^- = 1 \cdot 10^{-3}/s$ (faster unbinding), and $k_{cat} = 1 \cdot 10^{-3}/s$. As experiments in Fig. 2 of the manuscript show that 3 nM of gene are sufficient to release more than 30% of the inhibitor, and that addition of large amount of gene does not majorly change the *speed* of activation, we infer that the Michaelis-Menten constant of the enzyme $K_M = (k^- + k_{cat})/k^+$ is low, hence our choice of large k^+ when compared with the

literature[7,8]. From preliminary simulations, we estimated the concentration of $RNAP$ to be $150\,nM$ when supplying 4 units of enzyme, which is not too far from previous estimates[7,8]. This concentration was scaled linearly when less units were added in Fig. 2 of the manuscript. However, the experiments reported in Supplementary Figure 32 show slow kinetics of activation when compared to the gene variation experiments in Supplementary Figure 31; these results could be explained only by assuming a less concentrated and less efficient batch of enzymes, thus we assumed that 4 units correspond to $75\,nM$ and $k^+ = 0.5 \cdot 10^6/M/s$. Where multiple genes are present, we assume the enzyme binding/unbinding and catalytic activity are the same, and that the genes compete for enzyme binding.

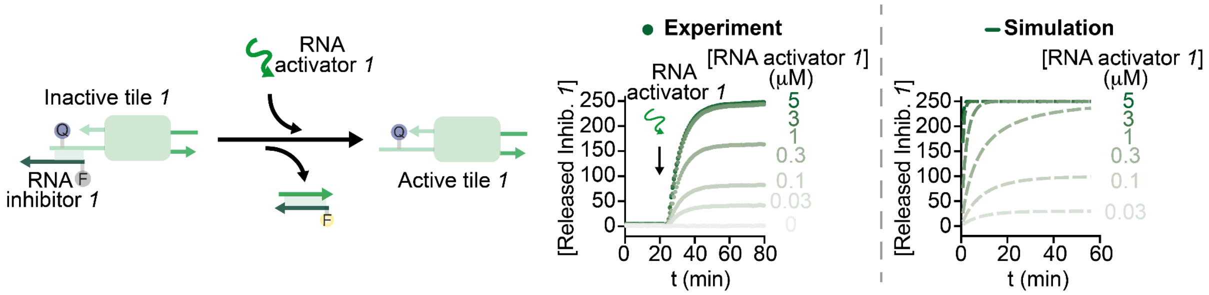

Supplementary Figure 39. *Tile 1 activation using synthetic RNA activator 1 supplied manually. Left: schematic of the reaction. Right: Experimental results and simulations with reaction rates listed in the text. Experiments were performed at 30° C in 1X transcription buffer, 10 mM NTPs, pH 8.0 in a 100 µL cuvette. Experimental values are averages of three separate measurements and error bars reflect standard deviations.*

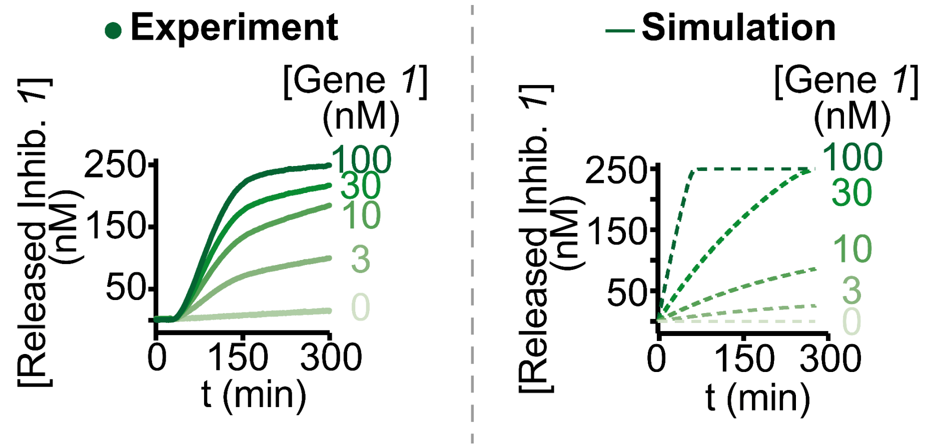

Supplementary Figure 40. *Comparison of experiments and simulation results for inhibitor release when changing the amount of gene (Fig. 2c of the manuscript). Experiments were performed at 30° C, in 1X transcription buffer, 10 mM NTPs, pH 8.0 in a 100 µL cuvette. Experimental values are averages of three separate measurements and error bars reflect standard deviations.*

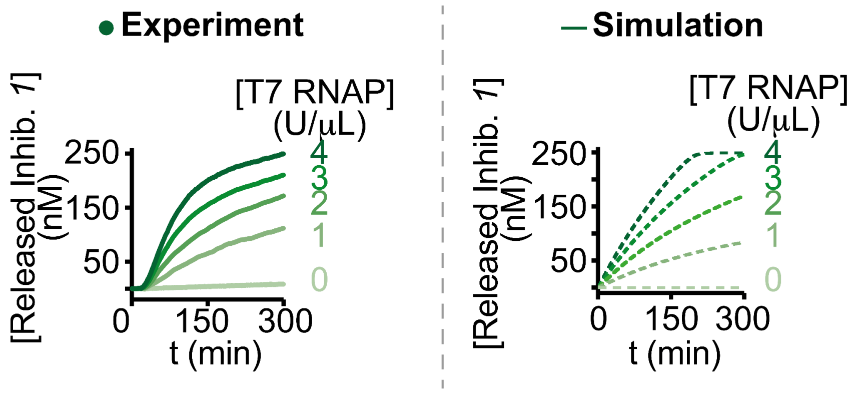

Supplementary Figure 41. *Comparison of experiments and simulation results for inhibitor release when changing the amount of T7 RNAP supplied to the system (Fig. 2e of the manuscript). Experiments were performed at 30° C, in 1X transcription buffer, 10 mM NTPs, pH 8.0 in a 100 µL cuvette. Experimental values are averages of three separate measurements and error bars reflect standard deviations.*

Supplementary References

1. Owczarzy, R. *et al.* IDT SciTools: a suite for analysis and design of nucleic acid oligomers. *Nucleic Acids Res.* **36**, W163–9 (2008).
2. Zadeh, J. N. *et al.* NUPACK: Analysis and design of nucleic acid systems. *J. Comput. Chem.* **32**, 170–173 (2011).
3. Rothemund, P. W. K. *et al.* Design and characterization of programmable DNA nanotubes. *J. Am. Chem. Soc.* **126**, 16344–16352 (2004).
4. Zhang, D. Y., Hariadi, R. F., Choi, H. M. T. & Winfree, E. Integrating DNA strand-displacement circuitry with DNA tile self-assembly. *Nat. Commun.* **4**, 1965 (2013).
5. Franco, E. *et al.* Timing molecular motion and production with a synthetic transcriptional clock. *Proc. Natl. Acad. Sci. U. S. A.* **108**, E784–93 (2011).
6. Kim, J. & Winfree, E. Synthetic in vitro transcriptional oscillators. *Molecular Systems Biology* vol. 7 465 Preprint at https://doi.org/10.1038/msb.2010.119 (2011).
7. Weitz, M. *et al.* Diversity in the dynamical behaviour of a compartmentalized programmable biochemical oscillator. *Nat. Chem.* **6**, 295–302 (2014).
8. Green, L. N. *et al.* Autonomous dynamic control of DNA nanostructure self-assembly. *Nat. Chem.* **11**, 510–520 (2019).
